# R278H Substitution Reduces Mouse Tyrosine Hydroxylase Activity and Increases Susceptibility to Catechol Inhibition

**DOI:** 10.3390/ijms27209370

**Published:** 2026-10-21

**Authors:** Ismail Alsalloum, Ekatherine I. Terentieva, Vitalii S. Moskaliuk, Sophia M. Antonova, Tatiana V. Ilchibaeva, Alexander V. Kulikov

**Affiliations:** Institute of Cytology and Genetics, Siberian Branch of Russian Academy of Sciences, 630090 Novosibirsk, Russia; ismailalsalom7@gmail.com (I.A.); terentevaei@bionet.nsc.ru (E.I.T.); moskaliukvs@bionet.nsc.ru (V.S.M.); antonovasm@bionet.nsc.ru (S.M.A.); ilchibaeva@bionet.nsc.ru (T.V.I.)

**Keywords:** tyrosine hydroxylase, recombinant protein, missense mutation, enzyme kinetics, thermal stability, differential scanning fluorimetry, catechol inhibition, mouse

## Abstract

Tyrosine hydroxylase (TH) catalyzes the hydroxylation of L-tyrosine to L-3,4-dihydroxyphenylalanine (L-DOPA), which is the rate-limiting step in dopamine (DA) synthesis in the brain. Certain mutations in the human *TH* gene are associated with severe neurological and psychiatric disorders. The c.886C>T mutation in the mouse *Th* gene, which results in the R278H substitution, decreases TH activity in the mouse midbrain. Here, we compared the molecular characteristics of the wild-type (TH-278R) and mutant (TH-278H) recombinant forms of TH. Both recombinant proteins were expressed in *Escherichia coli* BL21 cells and purified by immobilized metal affinity chromatography using Ni-NTA agarose resin. TH activity was assayed by HPLC, and the thermal stability of the recombinant proteins was assessed by differential scanning fluorimetry. The R278H substitution markedly decreased *V_max_* and increased the *K_M_* for the cofactor 6,7-dimethyltetrahydropterin (DMPH_4_), but had no effect on the *K_M_* for the substrate L-tyrosine. The substitution did not affect the *T*_50_ of thermal inactivation but markedly increased the *T_m_* of thermal unfolding of the TH-278H protein. The substitution decreased the *IC*_50_ values for dopamine and 1,2-dihydroxybenzene several-fold, both of which contain a catechol moiety that is also present in L-DOPA. These results indicate that the R278H substitution alters the sensitivity of TH to catechol-containing compounds. Although direct evidence is lacking, endogenous L-DOPA produced during the reaction may interact differently with the catalytic center of the TH-278H mutant than with that of the wild-type TH-278R.

## 1. Introduction

Tyrosine hydroxylase (TH) catalyzes the hydroxylation of L-tyrosine to L-3,4-dihydroxyphenylalanine (L-DOPA), the rate-limiting step in the synthesis of dopamine (DA) in the brain. Indeed, *Th* gene knockout reduces DA levels in the mouse brain [1,2]. Certain mutations in the human *TH* gene are associated with childhood parkinsonism [3], an increased risk of Parkinson’s disease [3,4], dystonia [5,6,7,8], and bipolar disorder [9]. However, investigating the molecular mechanisms linking *TH* gene mutations to neurological disorders, motor impairments, and alterations in human behavior and mental functions is constrained by social and ethical considerations. Thus, modeling the molecular disturbances caused by *Th* gene mutations in laboratory rodents represents an important challenge in modern neuroscience.

Among the missense variants in the mouse *Th* gene reported in the Ensembl genome database (https://www.ensembl.org/index.html (accessed on 20 September 2023)), the c.886C>T variant has been identified in laboratory mouse strains: the 886T allele is present in C57BL/6, C3H, DBA/2, and CBA mice, whereas the 886C allele is found in BALB/c and *Mus musculus castaneus* mice [10]. This mutation results in the R278H substitution in the mouse TH protein. The R278H substitution has functional consequences, including reduced enzyme activity and DA levels in the mouse midbrain [10,11], which increases the value of this mutation as a model for studying pathological conditions associated with TH deficiency. At the same time, the molecular mechanism underlying the reduced TH activity caused by this substitution remains unclear.

Theoretically, the R278H substitution could reduce the functional efficiency of the catalytic center by altering substrate and/or cofactor interactions, or decrease the stability of the enzyme molecule. Moreover, because the catechol moiety of L-DOPA can potentially chelate the iron ion in the catalytic center of the enzyme [12,13], the substitution could alter the interaction of the enzyme with its product, L-DOPA, and thereby potentially affect product-mediated inhibition.

The main aim of the present study was to investigate three possible factors contributing to the reduced TH activity associated with the R278H substitution: altered substrate/cofactor interactions, altered protein stability, and potential effects of the enzyme product, L-DOPA. For this purpose, we expressed, extracted, and purified recombinant TH-278R and TH-278H forms of mouse TH and compared their (1) *V_max_* and *K_M_* values for both the substrate and cofactor, (2) thermal stability of catalytic activity (*T*_50_) and thermal unfolding of the protein (*T_m_*), and (3) *IC*_50_ values for two catechol-containing compounds, dopamine and 1,2-dihydroxybenzene, as probes of the interaction of TH with catechol-containing molecules.

## 2. Results

### 2.1. Electrophoretic Mobility During SDS-PAGE

**Figure 1:**
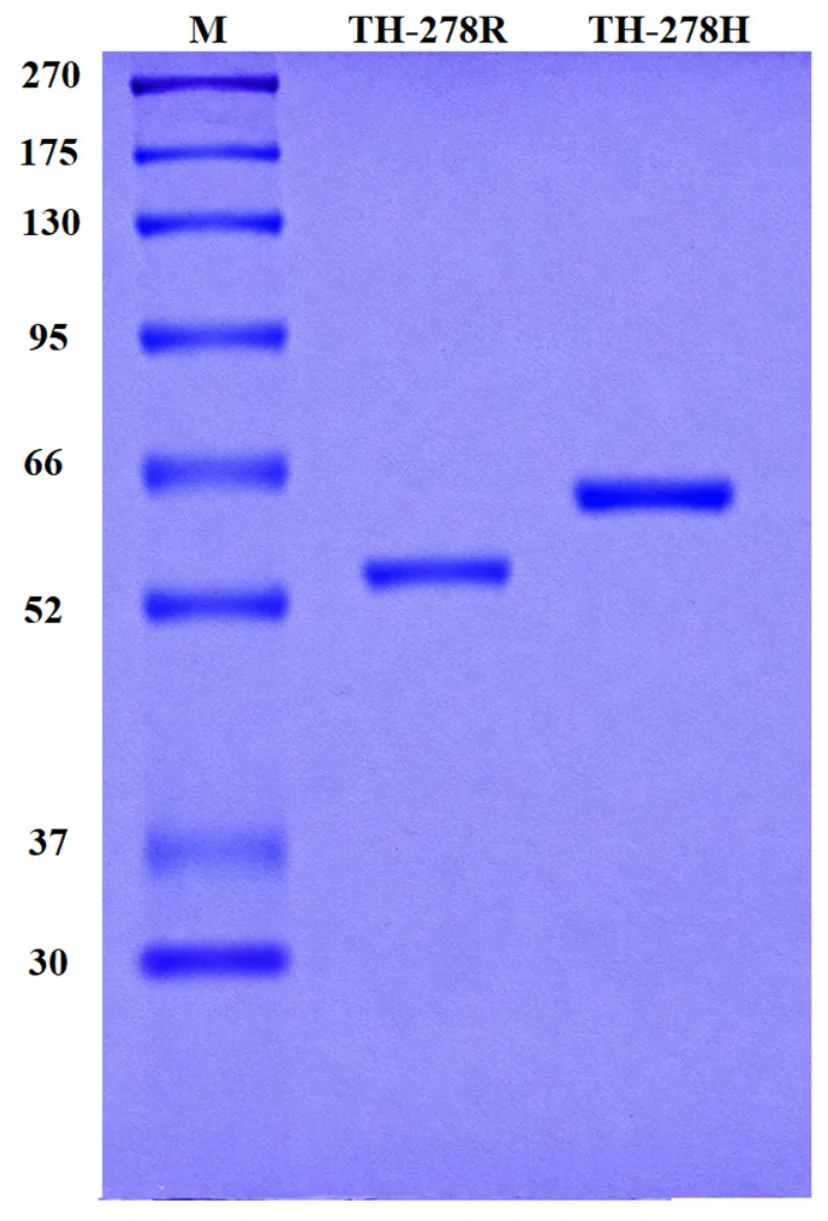
SDS-PAGE analysis of purified recombinant TH-278R and TH-278H forms of mouse TH (Coomassie staining).

The mutant recombinant protein (TH-278H) migrated more slowly during SDS-PAGE than the wild-type protein (TH-278R) (Figure 1).

### 2.2. Dependence of L-DOPA Synthesis on the Amount of Protein and Incubation Time

L-DOPA synthesis demonstrated a strong linear relationship with the amount of TH-278R protein (R^2^ = 0.991, *n* = 24) and TH-278H protein (R^2^ = 0.988, *n* = 24) during a 15 min incubation (Figure S1, Supplementary Materials). This confirms that activity measurements were performed within the linear range with respect to protein concentration.

Similarly, L-DOPA synthesis increased linearly with incubation time within the first minute for both TH-278R (R^2^ = 0.997, *n* = 12) and TH-278H (R^2^ = 0.993, *n* = 12) (Figure S2, Supplementary Materials). Based on this observation, a 1 min incubation was selected for the saturation experiments to determine *V_max_* and *K_M_* (Section 2.3).

During prolonged incubation, the rate of L-DOPA synthesis by both TH-278R and TH-278H gradually declined, possibly due to enzyme degradation (Figure S3, Supplementary Materials).

### 2.3. K_M_ and V_max_ Values for the Cofactor and L-Tyrosine

**Figure 2:**
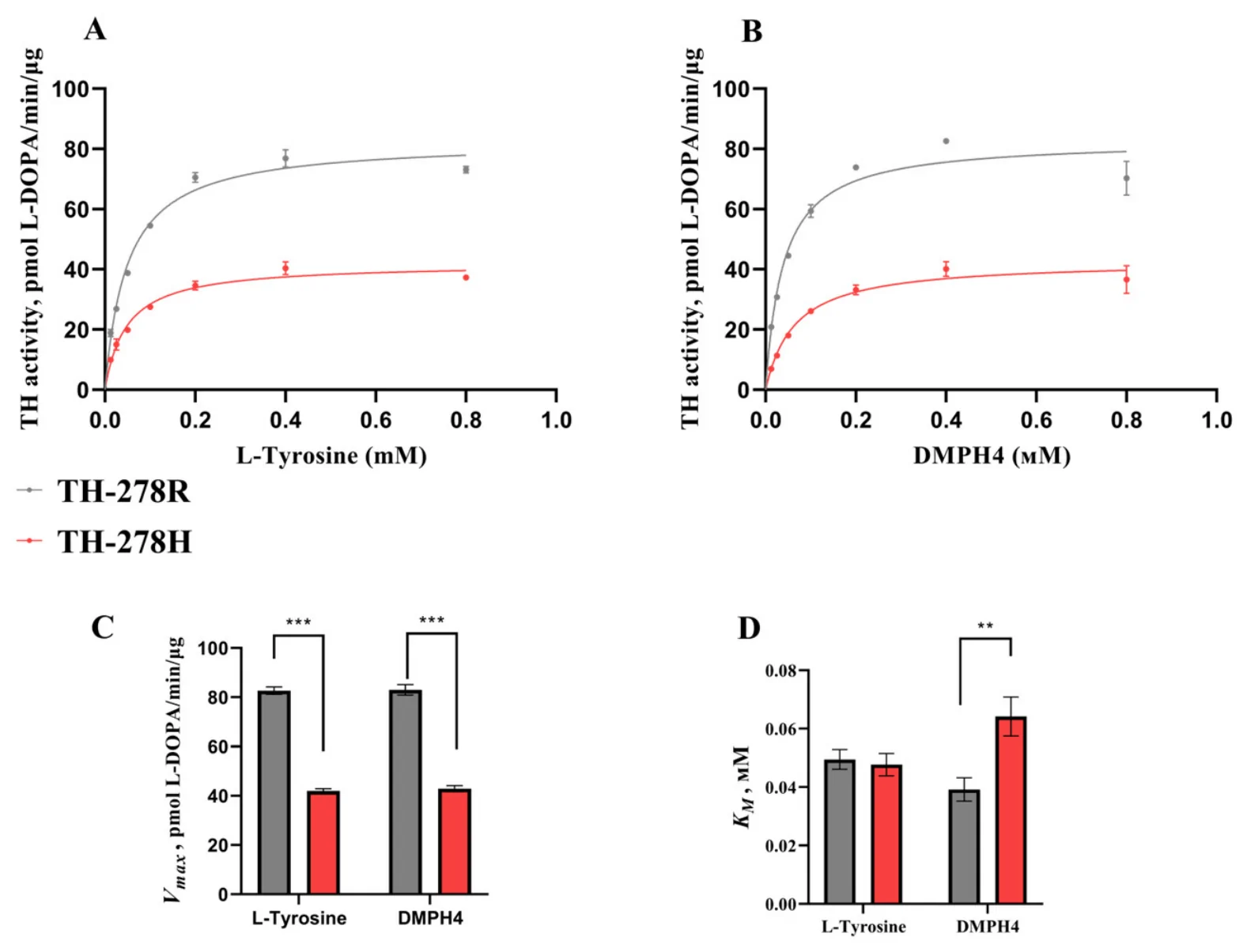
Kinetic parameters of the recombinant TH-278R and TH-278H forms of TH. (**A**) Dependence of TH activity on L-tyrosine concentration at a fixed DMPH_4_ concentration of 0.3 mM. (**B**) Dependence of TH activity on DMPH_4_ concentration at a fixed L-tyrosine concentration of 0.3 mM. In (**A**,**B**), solid lines represent the fit to the Michaelis–Menten equation for TH-278R and TH-278H. (**C**) *V_max_* for L-tyrosine and DMPH_4_. (**D**) *K_M_* for L-tyrosine and DMPH_4_. Mean ± SEM (*n* = 4) are presented. Incubation time was 1 min. ** *p* < 0.01, *** *p* < 0.001.

Representative Michaelis–Menten curves for TH-278R and TH-278H were generated using L-tyrosine (Figure 2A; TH-278R: R^2^ = 0.9771, *n* = 28; TH-278H: R^2^ = 0.9666, *n* = 28) and DMPH_4_ (Figure 2B; TH-278R: R^2^ = 0.9439, *n* = 28; TH-278H: R^2^ = 0.9561, *n* = 28) as substrates. Analysis of these curves yielded the *V_max_* and *K_M_* values summarized in Figure 2C and Figure 2D, respectively.

*V_max_* values (pmol L-DOPA/min/µg protein) were significantly higher for TH-278R than for TH-278H when determined from the L-tyrosine concentration series (82.71 ± 1.49 vs. 41.96 ± 0.90; t(54) = 23.43, *p* < 0.001) and from the DMPH_4_ concentration series (83.00 ± 2.14 vs. 42.85 ± 1.27; t(54) = 16.16, *p* < 0.001) (Figure 2C).

Regarding *K_M_*, the R278H substitution did not significantly affect the *K_M_* for L-tyrosine (0.0494 ± 0.003 vs. 0.0476 ± 0.003 mM; t(54) = 0.448, *p* = 0.656) but significantly increased the *K_M_* for the cofactor DMPH_4_ (0.0392 ± 0.004 vs. 0.0641 ± 0.006 mM; t(54) = 3.21, *p* = 0.0022) (Figure 2D).

### 2.4. Effect of Preincubation Temperature on TH Activity

**Figure 3:**
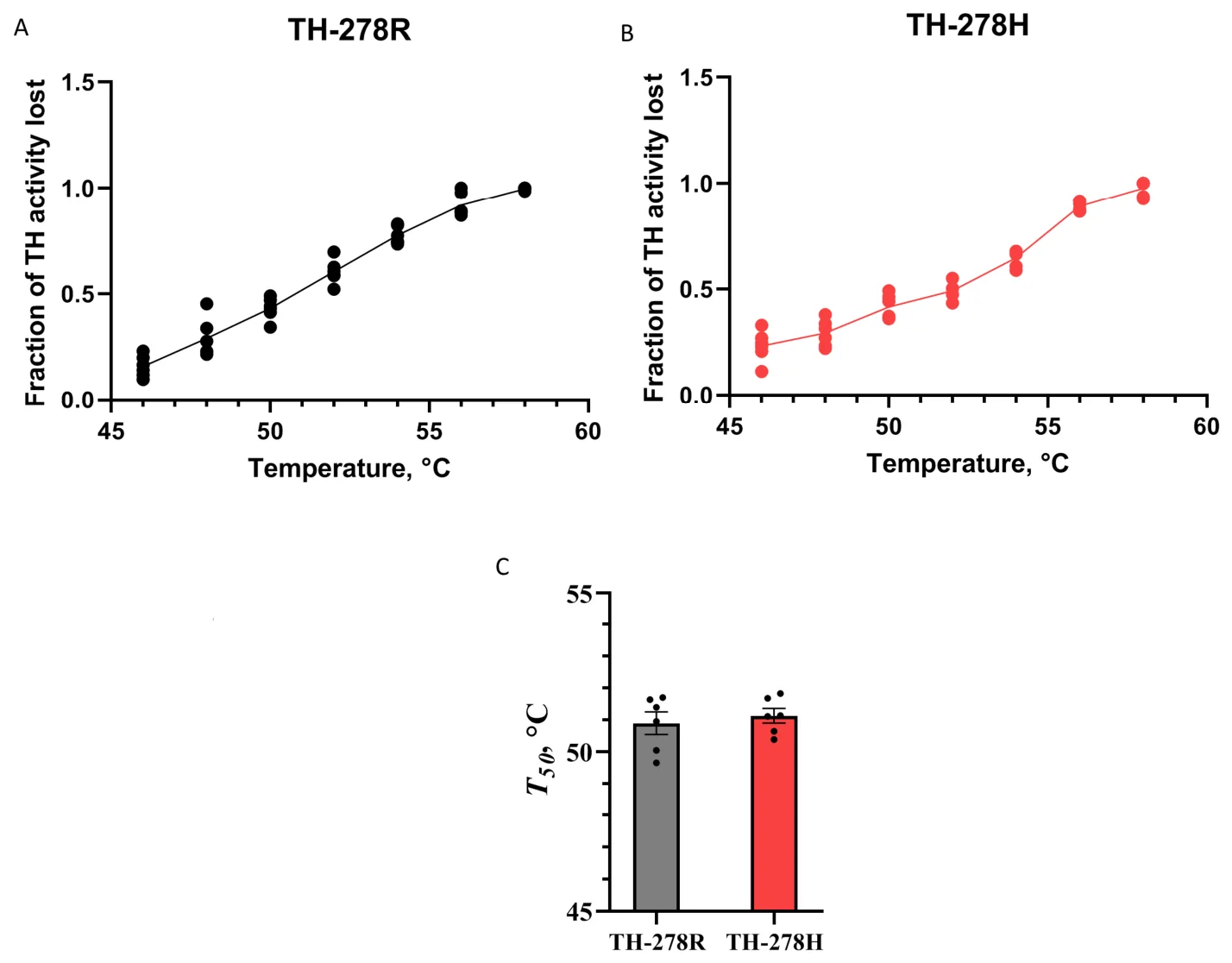
Effect of preincubation temperature on the activity of the TH-278R and TH-278H forms of TH. (**A**) Dependence of the fraction of TH-278R activity lost on preincubation temperature. (**B**) Dependence of the fraction of TH-278H activity lost on preincubation temperature. (**C**) *T*_50_ values for the TH-278R and TH-278H forms. Mean ± SEM (*n* = 6) are presented.

The effect of preincubation temperature on TH activity was evaluated using *T*_50_, defined as the temperature at which 50% of the initial enzyme activity was lost after preincubation. One-way ANOVA revealed no significant effect of the R278H substitution on *T*_50_ (TH-278R, 50.90 ± 0.35 °C; TH-278H, 51.13 ± 0.23 °C; F(1,10) < 1) (Figure 3).

### 2.5. Thermal Unfolding

**Figure 4:**
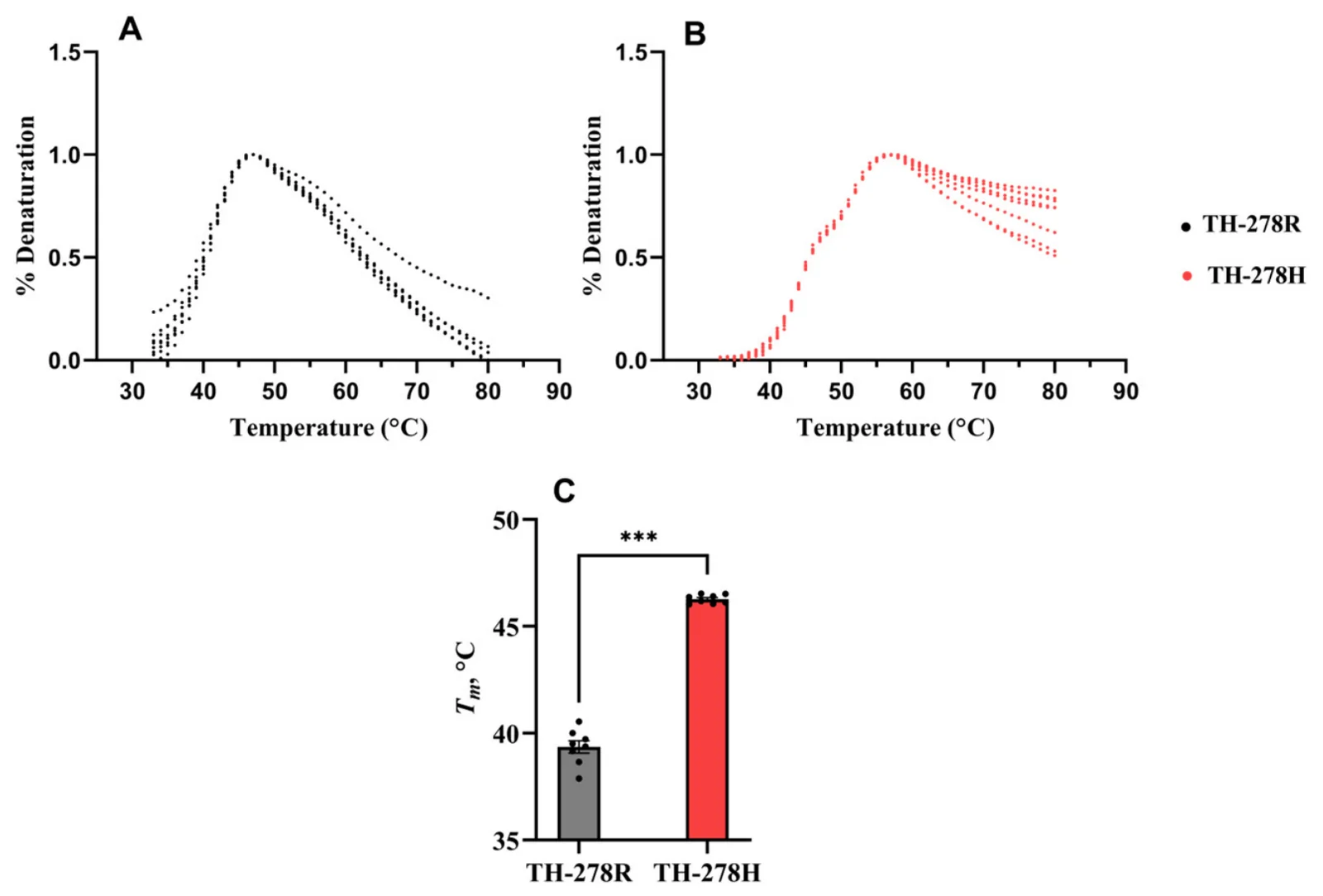
Thermal unfolding curves of the recombinant TH-278R and TH-278H forms of TH, measured by differential scanning fluorimetry (DSF). (**A**) TH-278R unfolding curves. (**B**) TH-278H unfolding curves. (**C**) *T_m_* values for the TH-278R and TH-278H forms. Mean ± SEM (*n* = 8) are shown. *** *p* < 0.001.

Thermal unfolding of the protein molecule was assessed by determining its melting temperature (*T_m_*), defined as the temperature at which 50% of the molecules are unfolded. The *T_m_* value for TH-278H (46.27 ± 0.07 °C) was significantly higher than that for TH-278R (39.36 ± 0.29 °C; F(1,14) = 536.45; *p* < 0.001) (Figure 4). Thus, the R278H substitution increased the *T_m_* of the TH molecule by 6.9 °C, indicating greater thermal stability of the TH-278H form than that of the TH-278R form.

### 2.6. Inhibition of TH Activity by Dopamine

**Figure 5:**
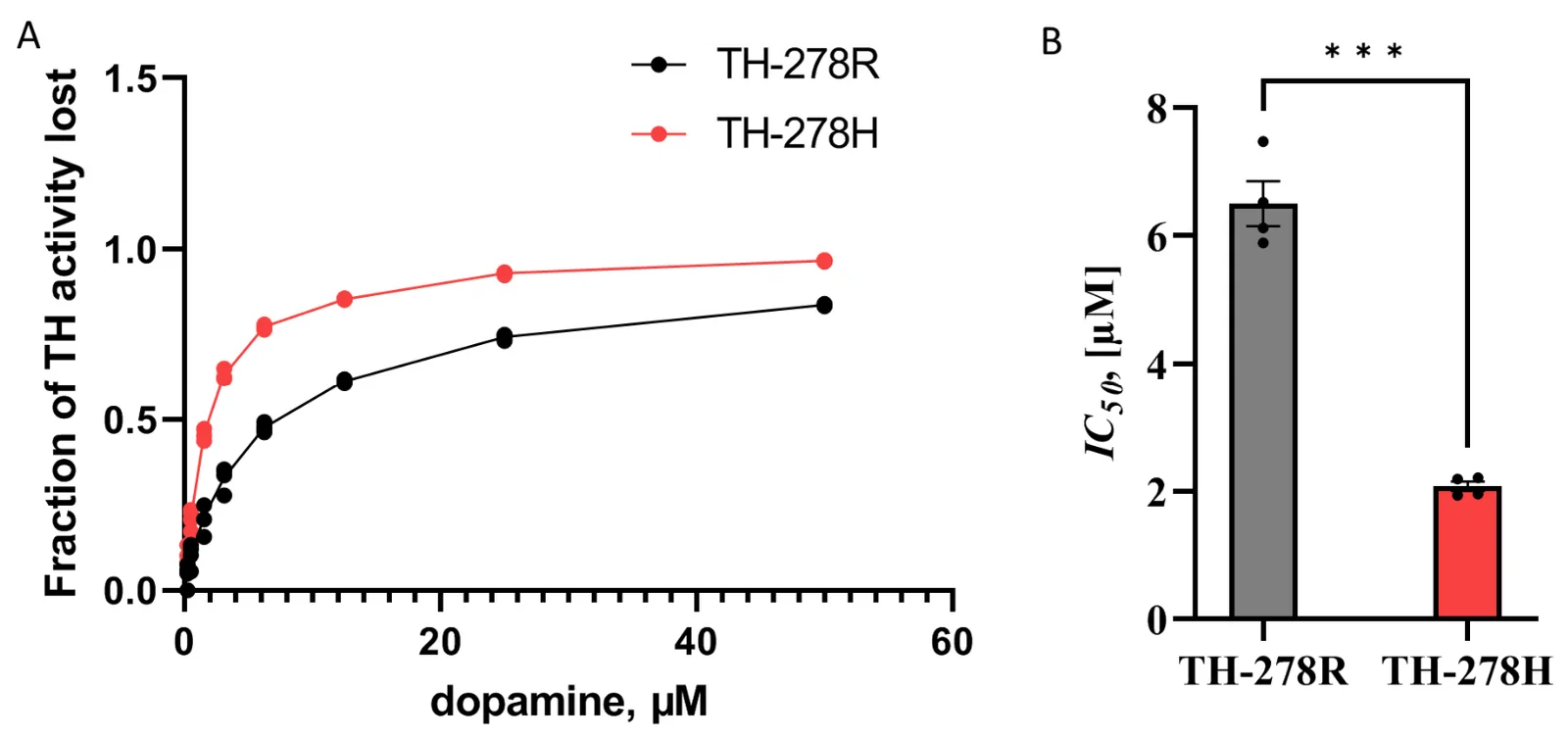
Inhibition of recombinant TH-278R and TH-278H by dopamine. (**A**) Dependence of the fraction of TH activity lost on dopamine concentration for TH-278R and TH-278H. (**B**) *IC*_50_ values for dopamine for TH-278R and TH-278H (mean ± SEM; *n* = 4). *** *p* < 0.001.

The ability of dopamine to inhibit TH was assessed by determining the *IC*_50_, defined as the dopamine concentration that caused a 50% reduction in enzyme activity. The *IC*_50_ value for dopamine was significantly lower for TH-278H (2.07 ± 0.07 μM) than for TH-278R (6.50 ± 0.35 μM; F(1,6) = 154.56; *p* < 0.001) (Figure 5).

### 2.7. Inhibition of TH Activity by 1,2-Dihydroxybenzene

**Figure 6:**
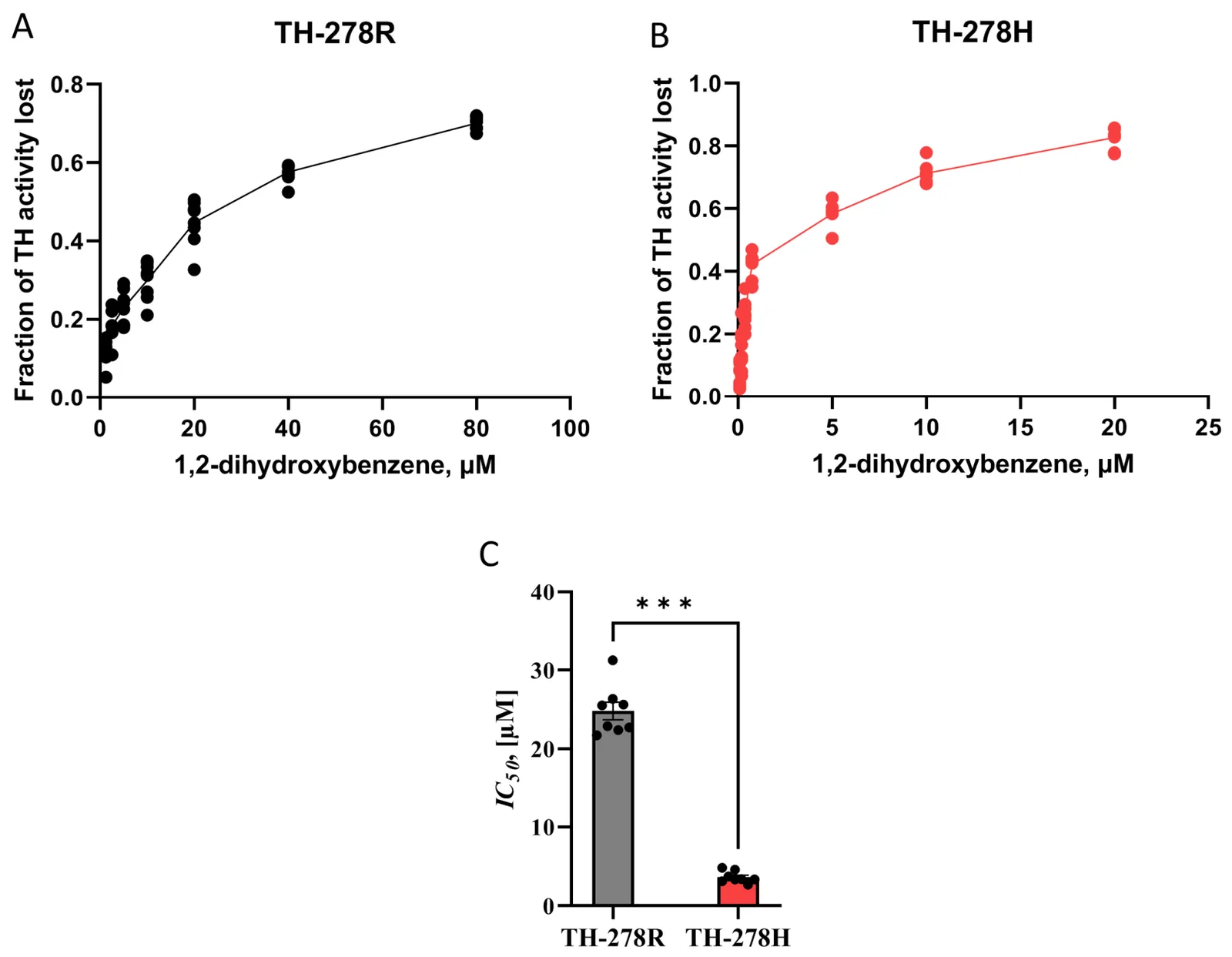
Inhibition of recombinant TH-278R and TH-278H by 1,2-dihydroxybenzene. (**A**,**B**) Dependence of the fraction of TH activity lost on 1,2-dihydroxybenzene concentration for TH-278R and TH-278H, respectively. (**C**) *IC*_50_ values for 1,2-dihydroxybenzene for TH-278R and TH-278H. Mean ± SEM (*n* = 8) are shown. *** *p* < 0.001.

The ability of 1,2-dihydroxybenzene to inhibit TH was assessed by determining the *IC*_50_, defined as the 1,2-dihydroxybenzene concentration that caused a 50% reduction in enzyme activity. The *IC*_50_ value for 1,2-dihydroxybenzene was significantly lower for TH-278H (3.63 ± 0.26 μM) than for TH-278R (24.80 ± 1.11 μM; F(1,14) = 347.3; *p* < 0.001) (Figure 6).

## 3. Discussion

It has previously been shown that the R278H substitution reduces TH activity in the mouse midbrain [10,11], although the molecular mechanism underlying this reduction has remained unknown. The first step in addressing this question was the expression and purification of recombinant TH-278R and TH-278H proteins. For this purpose, the plasmids pMAL-c2-10xHis-TEV-TH-278R and pMAL-c2-10xHis-TEV-TH-278H, encoding the TH-278R and TH-278H forms of TH and codon-optimized for expression in *E. coli*, were constructed. The constructs were expressed in *E. coli*, as MBP-TH fusion proteins, which were purified by immobilized metal affinity chromatography (IMAC). The fusion proteins were then cleaved with Tobacco Etch Virus (TEV) protease, and the recombinant TH-278R and TH-278H proteins were purified by a second round of IMAC. The purity of the recombinant TH forms was verified by SDS-PAGE analysis, which showed a single band corresponding to the recombinant TH protein (Figure 1).

The R278H substitution decreased the electrophoretic mobility of the mutant protein during SDS-PAGE. This finding is consistent with the results of Shi et al. [14], who showed that the R80H substitution decreased the electrophoretic mobility of recombinant human hSOD1 in SDS-PAGE. These authors hypothesized that some amino acid substitutions may alter SDS binding and thereby affect the electrophoretic mobility of proteins [14].

The kinetic parameters *V_max_* and *K_M_* values for L-tyrosine and DMPH_4_ were determined for the recombinant TH-278H and TH-278R forms. The two enzyme forms did not differ significantly in *K_M_* for L-tyrosine. However, the *K_M_* for DMPH_4_ was higher for the mutant TH-278H than for the wild-type TH-278R form. In addition, the *V_max_* of the recombinant TH-278H form was lower than that of TH-278R for both L-tyrosine and DMPH_4_. These findings are consistent with and provide a molecular characterization of the decreased activity previously reported for the mutant enzyme in the mouse midbrain [10,11].

From a mechanistic perspective, the R278H substitution could reduce the activity of recombinant TH through two possible mechanisms: (1) altered stability of the mutant protein and (2) altered interaction of the mutant catalytic center with its product, L-DOPA.

Some amino acid substitutions disrupt protein folding, thereby decreasing protein stability and functional activity. Misfolding of an enzyme can result in a reduction in *V_max_*. Indeed, substitutions such as R202H in human TH1 [15], P447R in mouse TPH2 (tryptophan hydroxylase 2) [16], and R252W [17] and V106A [18] in human PAH (phenylalanine hydroxylase) have been reported to disrupt protein folding and substantially reduce the *V_max_* of the corresponding mutant enzymes. Protein stability is often assessed by measuring thermal stability, including the melting temperature (*T_m_*), which is commonly defined as the temperature at which 50% of the molecules are unfolded [15]. *T_m_* can be determined from thermal unfolding curves obtained using fluorescent probes such as ANS (8-anilinonaphthalene-1-sulfonic acid), whose fluorescence increases upon binding to hydrophobic regions exposed during protein unfolding [15,16,17,18]. However, ANS has an excitation wavelength of approximately 395 nm, and its use in thermal unfolding assays requires a suitable fluorescence spectrometer. By contrast, SYPRO Orange is widely used for thermal unfolding assays because its excitation and emission wavelengths are compatible with standard real-time PCR instruments. Using SYPRO Orange, thermal unfolding curves can therefore be obtained with a standard real-time PCR instrument [19,20,21]. In the present study, we used ProteOrange, a modified form of SYPRO Orange, and found that it effectively detected thermal unfolding of the recombinant TH proteins.

Unexpectedly, the *T_m_* of the mutant TH-278H form was 6.9 °C higher than that of the wild-type TH-278R form, indicating greater resistance to thermal unfolding under the experimental conditions. Analysis of the thermal unfolding profiles revealed an additional qualitative difference: at 80 °C, the fluorescence signal of the TH-278R–ProteOrange complex decreased to approximately zero, whereas that of the TH-278H–ProteOrange complex remained at approximately 50% of its maximal value. This residual fluorescence may reflect thermally induced aggregation accompanied by retention or trapping of ProteOrange within protein aggregates. Thus, the thermal unfolding data do not support the hypothesis that the R278H substitution decreases the intrinsic thermal stability of the TH protein. Rather, they indicate that the TH-278H form is more resistant to thermal unfolding than TH-278R under the conditions tested.

Previously, we developed another model for assessing thermal stability based on measuring enzyme activity after 2 min of heating at 48–64 °C [16]. In the present study, both enzyme forms were heated at 46–58 °C. No significant difference in *T*_50_ was observed between the TH-278R and TH-278H forms. Taken together with the higher *T_m_* of TH-278H, these findings can be considered in the context of the stability–activity trade-off concept [22]. We suggest that increased structural stability of the TH-278H molecule may limit the conformational flexibility of the catalytic domain required for optimal positioning of the cofactor. Such a change could contribute to the observed increase in *K_M_* for DMPH_4_.

An important feature of TH is that the catechol moiety of its product, L-DOPA, can theoretically chelate the iron ion in the catalytic center [12,13], potentially complicating product release from the active site. Indeed, dopamine, which contains the same catechol moiety as L-DOPA, has been reported to strongly inhibit TH activity [23,24,25,26]. We hypothesized that the R278H substitution might alter the interaction of endogenous L-DOPA with the iron in the catalytic center, thereby potentially slowing product release. However, modeling by Maass et al. suggested that L-DOPA cannot penetrate into the TH catalytic center because its carboxylate group is repelled by the negatively charged residue Asp425 [27]. Our experimental results are consistent with this observation, as exogenous L-DOPA did not inhibit either TH-278R or TH-278H even at a concentration of 25 µM (Figure S4, Supplementary Materials). These modeling and experimental findings indicate that exogenous L-DOPA is unlikely to inhibit TH by entering the catalytic center and chelating the iron ion under the conditions tested.

Dopamine and 1,2-dihydroxybenzene, which contain the catechol moiety of L-DOPA but lack its carboxylate group, can interact with the catalytic center and inhibit TH at low concentrations. In the present study, we found that the *IC*_50_ values of both dopamine and 1,2-dihydroxybenzene were several-fold lower for TH-278H than for TH-278R, indicating greater sensitivity of the mutant enzyme to inhibition by these compounds. These findings suggest that the R278H substitution may alter TH interactions with catechol-containing compounds, but whether this finding is relevant to endogenous L-DOPA remains unknown.

Previous studies have demonstrated the important role of the Ser40 residue in the mechanism of TH inhibition by dopamine: phosphorylation of this residue decreases the inhibitory effect of dopamine on the enzyme [24]. In the present study, our results suggest that the 278R/H site may influence the interaction of the TH catalytic center with certain catechol-containing compounds.

**Figure 7:**
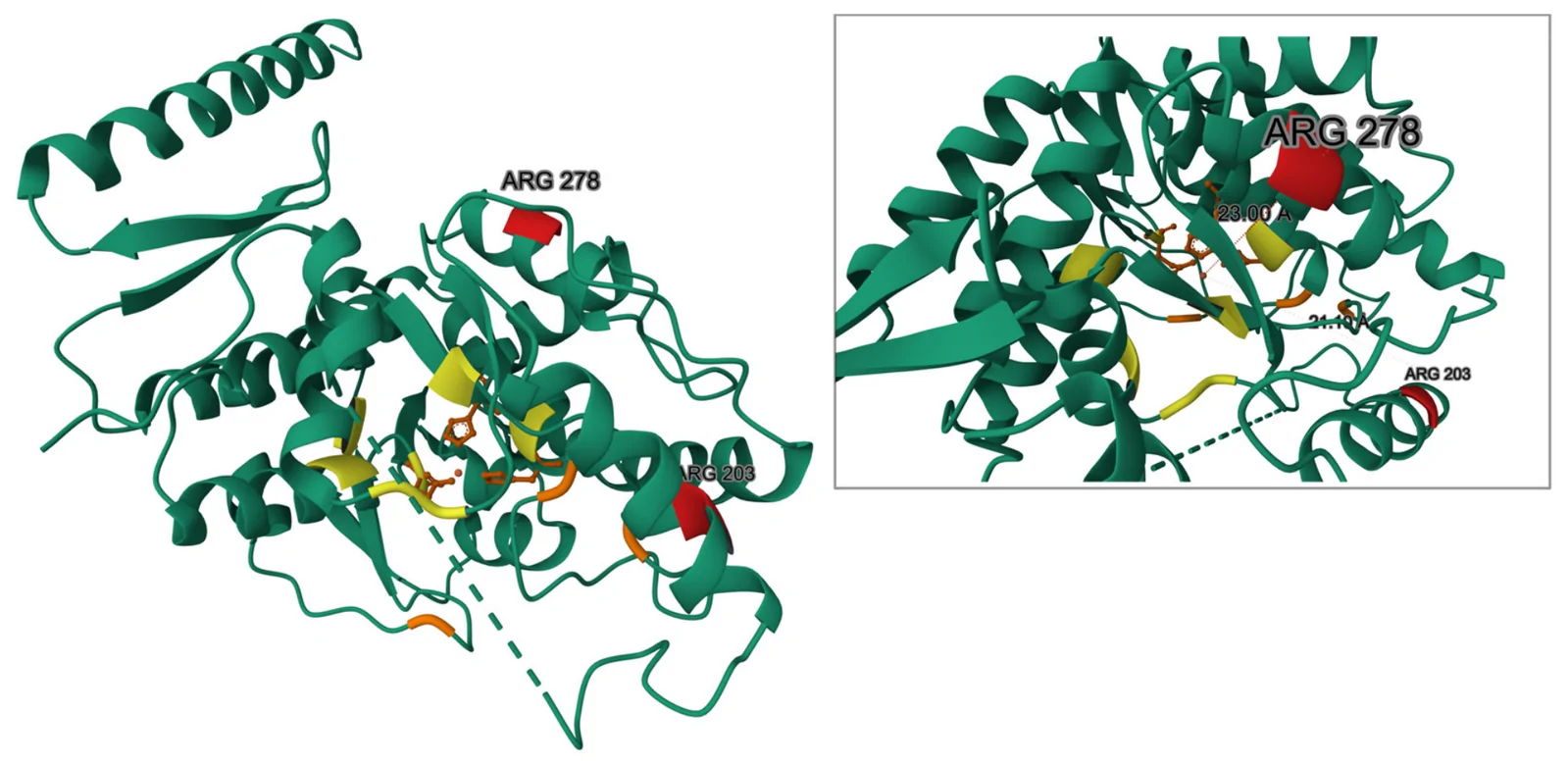
One subunit of the three-dimensional structure of rat TH (PDB ID: 1TOH; residues 162–498; Rattus norvegicus). Arg278 and Arg203 are shown in red, with their distances to the catalytic iron ion indicated (Arg278–Fe: 21.10 Å; Arg203–Fe: 23.00 Å); neither residue is directly involved in the active site. Residues coordinating the iron ion (His331, His336, and Glu376) are shown in ball-and-stick representation. Residues interacting with the BH_4_ cofactor (Leu294, Leu295, Phe300, Gln310, Glu332, Tyr371, and Glu376) are highlighted in yellow, whereas residues involved in L-tyrosine binding (Arg316, Asp328, and Asp425) are highlighted in orange. Visualization was performed using the Mol* Viewer (5.13.0) (RCSB PDB).

To visualize the spatial position of the 278R/H site relative to catalytically important residues, we used the crystal structure of rat TH (PDB ID: 1TOH; Figure 7). The use of the rat TH structure is justified by the high sequence identity between rat and mouse TH (97%). The structure contains residues that coordinate the Fe^2+^ ion (His331, His336, and Glu376), interact with the BH_4_ cofactor (Leu294, Leu295, Phe300, Gln310, Glu332, Tyr371, and Glu376), and participate in L-tyrosine binding (Arg316, Asp328, and Asp425) [28,29,30,31]. This analysis shows that Arg278 is not directly involved in the catalytic center and is located relatively far from the catalytic iron. These observations suggest that the R278H substitution may influence catalytic activity through changes in the local or long-range conformation of the protein rather than through direct participation in catalysis. Although the structural basis of this effect remains to be established, the R278H substitution may alter the three-dimensional environment of the catalytic center and thereby modify its interaction with catechol-containing molecules.

To further assess the functional significance of the R278H substitution, we compared this position with pathogenic mutations in the human *TH* gene. The adjacent residue at mouse position 279, corresponding to F309 in the human hTH4 isoform, is affected by the pathogenic F309S variant (c.926T>C) [32], for which enzyme activity has been reported to be no more than 0.2% of the wild-type level [33]. At mouse position 277, corresponding to S307 in the hTH4 isoform, the pathogenic S307C substitution has been associated with tyrosine hydroxylase deficiency (THD) and autism spectrum disorder [34]. The occurrence of clinically significant substitutions at residues surrounding position 278 suggests that this region of the TH molecule is functionally important.

The functional importance of arginine residues within the catalytic domain of TH is further supported by the findings of Körner et al. (2015), who generated knock-in mice carrying the R203H mutation, corresponding to the pathogenic human R233H variant [35]. These animals exhibited progressive depletion of central catecholamines, hypokinesia, and catalepsy. The R203H substitution was reported to alter substrate specificity, increase the *K_M_* for L-tyrosine, and reduce the solubility and thermal stability of the protein [34]. Notably, R203, like R278, is not directly involved in the active site, and the distances from these residues to the catalytic iron are comparable (21.10 Å and 23.00 Å, respectively; Figure 7). Nevertheless, the available data suggest that substitutions at these two positions appear to affect TH function through different mechanisms. R203H has been associated with impaired substrate binding and reduced protein stability, whereas R278H is associated with greater resistance to thermal unfolding, an increased *K_M_* for the DMPH_4_ cofactor, and lower *IC*_50_ values for the two catechol-containing compounds dopamine and 1,2-dihydroxybenzene.

It should also be noted that the C57BL/6 mouse strain carries the 278H allele of *Th*, which is associated with reduced TH activity and decreased dopamine levels in the midbrain [11]. The C57BL/6 strain is widely used as a genetic background for many transgenic and knockout mouse models. Therefore, neurobiological and behavioral findings obtained using C57BL/6 mice, as well as transgenic strains maintained on a C57BL/6 background, may need to be interpreted with this genetic feature in mind. This consideration may be particularly relevant for studies investigating the dopaminergic system or interventions targeting dopaminergic signaling.

## 4. Materials and Methods

### 4.1. Bacterial Strains and Plasmid Constructs

**Figure 8:**
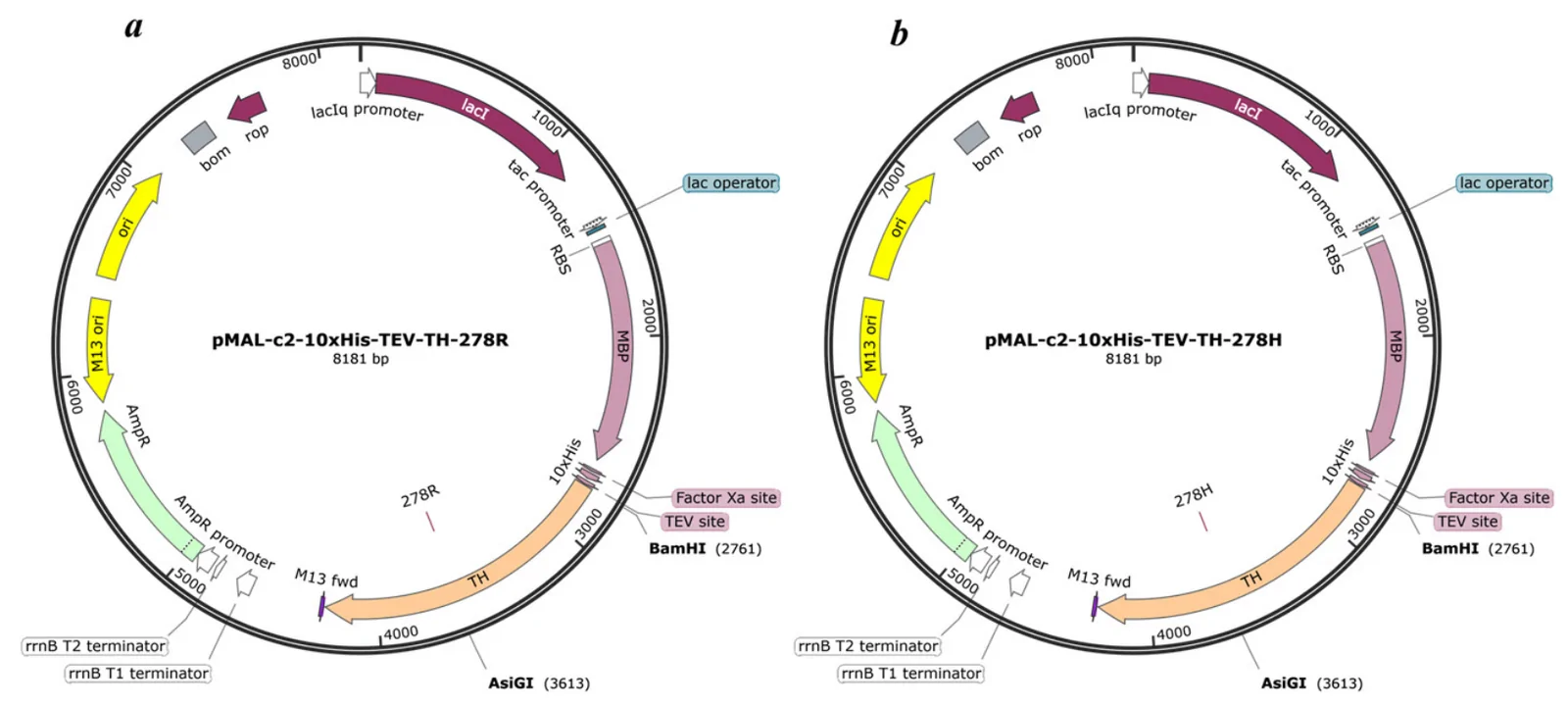
Plasmid constructs for expression of tyrosine hydroxylase. (**a**,**b**) Schematic maps of vectors pMAL-c2-10xHis-TEV-TH-278R and pMAL-c2-10xHis-TEV-TH-278H, respectively.

Two *Escherichia coli* strains were used: DH5α for plasmid cloning and BL21 for recombinant protein expression. Tyrosine hydroxylase was expressed using two plasmid constructs, pMAL-c2-10xHis-TEV-TH-278R and pMAL-c2-10xHis-TEV-TH-278H, encoding TH variants carrying Arg278 and His278, respectively. The coding sequence of the mouse (*Mus musculus*) *Th* gene was codon-optimized for expression in *E. coli* using the IDT Codon Optimization Tool. Both constructs (Figure 8a,b) were synthesized by Synbio Technologies (Suzhou, China) (China) and verified by Sanger sequencing.

All solutions were prepared in Milli-Q water and sterilized either by autoclaving at 121 °C for 20 min or by filtration through 0.22 µm membrane filters. Reagents were of analytical grade. All manipulations were performed under aseptic conditions adjacent to a Phoenix II gas burner (Schuett-biotec, Göttingen, Germany).

### 4.2. Preparation of Competent Escherichia coli Cells

Chemically competent *E. coli* cells (strains DH5α and BL21) were prepared using transformation buffer (TB) consisting of 10 mM PIPES, 15 mM CaCl_2_, 250 mM KCl, and 55 mM MnCl_2_ (pH 6.7). PIPES, CaCl_2_, and KCl were dissolved in ultrapure water, the pH was adjusted to 6.7 with NaOH, MnCl_2_ was added, and the buffer was sterilized by filtration through a 0.22 µm membrane filter.

A small aliquot of cells from the glycerol stock was inoculated into 4 mL of 2×YT medium (Carl Roth GmbH + Co KG, Karlsruhe, Germany) and cultured overnight at 37 °C with shaking at 240 rpm in an AG CH-4103 shaker incubator (INFORS HT, Bottmingen, Switzerland). The overnight culture was diluted 1:100 into SOB medium (20 g/L tryptone, 5 g/L yeast extract, 8.6 mM NaCl, 2.5 mM KCl, supplemented after autoclaving with sterile MgCl_2_ and MgSO_4_ to final concentrations of 10 mM each) and incubated at 37 °C with shaking at 180 rpm for 2–3 h. Growth was monitored by measuring optical density at 600 nm (OD_600_; BioPhotometer Plus, Eppendorf, Hamburg, Germany); incubation was terminated at OD_600_ = 0.5 (mid-exponential phase).

The culture was chilled on ice for 20 min and centrifuged at 4000 rpm, 4 °C for 10 min (Allegra 25R, Beckman Coulter, Brea, CA, USA). The pellet was resuspended in 30 mL of ice-cold TB and incubated on ice for 30 min, after which the cells were centrifuged again under the same conditions. The pellet was resuspended in 10 mL TB, dimethyl sulfoxide (DMSO) was added to a final concentration of 7% (*v*/*v*), and the suspension was incubated on ice for 10 min. Competent cells were aliquoted, snap-frozen in liquid nitrogen, and stored at −80 °C.

### 4.3. Plasmid Transformation

Plasmid DNA was introduced into competent *E. coli* cells by heat shock. One microliter of plasmid DNA (50–100 ng) was added to 100 µL of competent cells and mixed gently. Controls included cells without plasmid and a reference plasmid with a confirmed transformation efficiency. The mixture was incubated on ice for 30 min, heat-shocked at 42 °C for 90 s, and immediately transferred to ice for 2 min. Cells were recovered by adding 800 µL of 2×YT medium and incubating at 37 °C with shaking at 240 rpm for 1 h. A 50 µL aliquot was plated on 2×YT agar supplemented with 150 µg/mL ampicillin (Carl Roth GmbH + Co KG, Karlsruhe, Germany) and incubated at 37 °C for 12–16 h until colonies appeared.

### 4.4. Plasmid Isolation and Verification

Individual colonies were inoculated into 2×YT medium with ampicillin (150 µg/mL) and cultured overnight at 37 °C, 240 rpm. Plasmid DNA was isolated using the FastPure EndoFree Plasmid Mini Kit (Vazyme, Nanjing, China) according to the manufacturer’s instructions.

**Figure 9:**
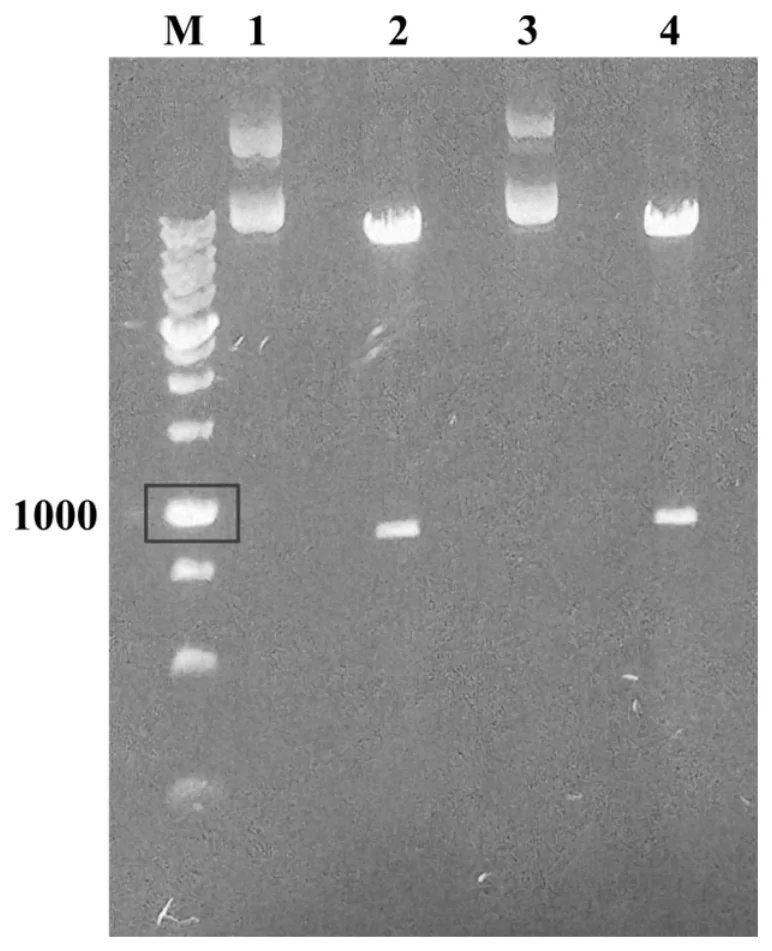
Restriction analysis of plasmids. M: DNA molecular weight marker M11 1 kb (SibEnzyme, Novosibirsk, Russia; 13 fragments ranging from 0.25 to 10.0 kbp; the 1000 bp fragment is indicated by a box); lane 1: pMAL-c2-10xHis-TEV-TH-278R (uncut plasmid DNA showing various topological forms); lane 2: pMAL-c2-10xHis-TEV-TH-278R restriction digest with BamHI and AsiGI (7329 and 852 bp); lane 3: pMAL-c2-10xHis-TEV-TH-278H (uncut plasmid DNA showing various topological forms); lane 4: pMAL-c2-10xHis-TEV-TH-278H restriction digest with BamHI and AsiGI (7329 and 852 bp).

Plasmid integrity was confirmed by restriction analysis using BamHI and AsiGI endonucleases (SibEnzyme, Novosibirsk, Russia). Digestion products were resolved by electrophoresis on a 1% agarose gel in 1×TBE buffer (89 mM Tris, 89 mM boric acid, 2 mM EDTA, pH 8.0) containing 0.5 µg/mL ethidium bromide. Samples were mixed with 6× loading buffer (60 mM Tris-HCl, pH 8.0; 60 mM EDTA; 0.4% Orange G; 15% Ficoll 400) before loading. Restriction digestion yielded two fragments of 7329 and 852 bp, consistent with the expected plasmid architecture (Figure 9).

### 4.5. Recombinant Protein Expression

Plasmid constructs pMAL-c2-10xHis-TEV-TH-278R and pMAL-c2-10xHis-TEV-TH-278H were transformed into *E. coli* BL21 cells as described above. Glycerol stocks were prepared by growing a single colony in 5 mL of 2×YT with ampicillin (150 µg/mL) at 37 °C, 240 rpm for 16 h; cells were pelleted at 3600× *g*, 4 °C for 10 min, resuspended in 250 µL of 2×YT, supplemented with 250 µL of 40% (*v*/*v*) glycerol, snap-frozen in liquid nitrogen, and stored at −80 °C. Plasmid integrity in glycerol stocks was confirmed by restriction analysis prior to expression.

For protein production, a small aliquot of cells from the glycerol stock was inoculated into 5 mL of 2×YT with ampicillin (150 µg/mL) and grown overnight at 37 °C, 240 rpm. This pre-culture was used to inoculate 600 mL of 2×YT with ampicillin (150 µg/mL) at a 1:100 ratio. Cells were grown at 37 °C, 180 rpm until OD_600_ reached 0.4 (approximately 1.5 h), at which point the temperature was gradually reduced to 25 °C and cultivation continued until an OD_600_ of 0.6. Protein expression was induced by simultaneous addition of IPTG (250 µM, SibEnzyme, Novosibirsk, Russia), glucose (0.2% *w*/*v*), and FeSO_4_ (100 µM). FeSO_4_ was added as an iron source during recombinant protein expression. Induction was carried out at 25 °C, 180 rpm for 20 h. Cells were harvested by centrifugation at 3600× *g* at 4 °C; the pellet (~5 g wet weight) was snap-frozen in liquid nitrogen and stored at −80 °C until purification. Each TH variant was expressed and purified once, and all experiments described in this study were performed using the corresponding protein preparation. Replicate measurements represent repeated measurements of the same protein preparation rather than independent protein preparations.

### 4.6. Protein Purification

Recombinant MBP-10xHis-TH-278R and MBP-10xHis-TH-278H fusion proteins were purified in two steps by immobilized metal affinity chromatography (IMAC) on Ni-NTA agarose resin (AbiFlow 100 Ni-NTA Agarose Resin, Abisense, Sochi, Russia) using a TiEC 16/30 column connected to an ACHROM BASE chromatography system (Taidu Biotech, Suzhou, China). All buffers were prepared and used at 4 °C. Lysis buffer contained 50 mM Tris-HCl (pH 7.5), 100 mM NaCl, and 20 mM imidazole, supplemented immediately before use with 0.5 mM DTT, 1 mM PMSF, 5 µM E-64, 5 µM leupeptin, and 2 µM pepstatin A.

Cell pellets were resuspended in 30 mL lysis buffer, supplemented with lysozyme (1 mg/mL), DNase I (5 unit/mL), and MgCl_2_ (5 mM), and incubated on ice for 1 h with intermittent mixing. Cells were further disrupted by sonication (22 kHz; 30 s pulse/100 s rest; 6–10 cycles) with continuous cooling in an ice bath. The lysate was centrifuged at 12,000× *g*, 4 °C for 30 min, and the supernatant was filtered through a 0.22 µm membrane filter.

The clarified lysate was loaded onto a Ni-NTA column pre-equilibrated with wash buffer (50 mM Tris-HCl, pH 7.5; 100 mM NaCl; 20 mM imidazole; 5 column volumes) at a flow rate of 3 mL/min. The column was washed with 20 column volumes of wash buffer at 5 mL/min until the absorbance at 280 nm returned to baseline. Bound protein was eluted stepwise with increasing imidazole concentrations; the main TH-containing fraction eluted at approximately 200 mM imidazole.

Imidazole was removed by buffer exchange using centrifugal concentrators (50 kDa molecular weight cut-off; Jet Biofil, Guangzhou, China) against cleavage buffer (50 mM Tris-HCl, pH 7.5; 100 mM NaCl; 1 mM DTT; 0.1% Triton X-100) by repeated concentration–dilution cycles. Protein concentration was determined by the Bradford assay and adjusted to approximately 3 mg/mL. The fusion protein was cleaved with TEV protease (Biolabmix, Novosibirsk, Russia) at a 1:100 enzyme-to-substrate mass ratio for 16 h at 4 °C with gentle agitation on an MR1 shaker (Biosan, Riga, Latvia). Cleavage efficiency was confirmed by SDS-PAGE.

Following cleavage, the sample was centrifuged at 3000× *g*, 4 °C for 20 min to remove insoluble aggregates, and subjected to a second round of Ni-NTA chromatography. The flow-through fraction containing purified TH was collected, while the MBP tag and TEV protease, both carrying His-tags, were retained on the resin and subsequently eluted with imidazole buffer. Final protein purity was confirmed by SDS-PAGE (Figure 1). Purified TH was supplemented with glycerol to 20% (*v*/*v*), aliquoted, snap-frozen in liquid nitrogen, and stored at −80 °C until use. The final concentrations of the TH-278R and TH-278H protein stocks were 0.64 and 0.5 mg/mL, respectively. Before experiments, these TH stocks were diluted 1:20 (32 and 25 µg/mL, respectively) with 50 mM Tris-HCl buffer (pH 7.6) containing 1 mM DTT. DTT was used to maintain reduced cysteine thiols and to minimize oxidative aggregation/inactivation of the purified enzyme during handling and dilution. It is a standard precaution for redox-sensitive metalloenzymes.

### 4.7. SDS-PAGE Analysis

For SDS-PAGE analysis, an equal volume of 2× Laemmli sample buffer (120 mM Tris-HCl, pH 6.8; 4% SDS; 20% glycerol; 0.02% bromophenol blue; 10% β-mercaptoethanol) was added to each sample. Samples were denatured at 95 °C for 5 min and then cooled on ice, followed by SDS-PAGE using running buffer containing 25 mM Tris, 192 mM glycine and 3.5 mM SDS, pH 8.3. Proteins were separated using stacking (4% acrylamide, 125 mM Tris-HCl, pH 6.8, 0.1% SDS, 0.1% APS and 0.1% TEMED) and resolving (10% acrylamide, 375 mM Tris-HCl, pH 8.8, 0.1% SDS, 0.1% APS and 0.1% TEMED) polyacrylamide gels. Electrophoresis was performed at 130 V until the marker exited the stacking gel, then at 230 V for 45 min. Gels were stained with Coomassie Brilliant Blue R-250 (Bio-Rad, Hercules, CA, USA) for 1 h and destained for 1 h.

### 4.8. Assay of TH Activity

The incubation mixture contained 15 µL of diluted TH corresponding to 0.48 and 0.375 µg of purified TH-278R and TH-278H protein, respectively, 0.3 mM L-tyrosine (Sigma-Aldrich, Steinheim, Germany), 0.3 mM 6,7-dimethyltetrahydropterin (DMPH_4_, Sigma-Aldrich, Germany), 5 U of catalase (Sigma-Aldrich, Germany), and 1 mM dithiothreitol (Sigma-Aldrich, Germany) in a final volume of 25 µL (50 mM Tris-HCl buffer, pH 7.6). Samples were incubated at 37 °C. The reaction terminated by adding 75 µL of 0.6 M HClO_4_ and then centrifuged at 12,700 rpm for 15 min. The clear supernatant was diluted twofold with ultrapure water. Synthesized L-DOPA was determined by HPLC on a Luna C18(2) column (100 mm × 4.6 mm; 5 µm particle size, Phenomenex, Torrance, CA, USA) equipped with a Zorbax SB-C8 guard column (12.5 mm × 4.6 mm; 5 µm particle size, Agilent, Santa Clara, CA, USA) using an LC-20AD chromatograph (Shimadzu Corporation, Kyoto, Japan). The mobile phase (pH 3.2) contained 13.06 g of KH_2_PO_4_, 200 µL of 0.5 M Na_2_EDTA, 300 mg of sodium 1-octanesulfonate, 940 µL of concentrated H_3_PO_4_, and 130 mL of methanol (13%) per liter. The mobile phase flow rate was 0.6 mL/min. The concentration of synthesized L-DOPA was determined using a DECADE II™ electrochemical detector and a VT-03 glassy carbon electrode (3 mm; Antec, Netherlands). The column and detector temperatures were maintained at 4 °C. Under these conditions, the retention time of L-DOPA on the column was 3.5 min. The area of the L-DOPA peak was determined using LabSolution LC/GC v. 5.54 software (Shimadzu Corporation, Japan), with L-DOPA standards at concentrations of 25, 50, and 100 pmol. TH activity was expressed as pmol of L-DOPA synthesized per minute per µg of protein.

Note: Because the retention time of L-DOPA coincided with those of 5,6,7,8-tetrahydrobiopterin and 6-methyl-5,6,7,8-tetrahydropterin, only DMPH_4_ was used as the cofactor.

### 4.9. Assay of the K_M_ and V_max_ of TH

To determine the *K_M_* and *V_max_* values for DMPH_4_, a 10 µL aliquot of diluted TH solution was incubated for 1 min at 37 °C with 0.3 mM L-tyrosine and DMPH_4_ at concentrations of 0.8, 0.4, 0.2, 0.1, 0.05, 0.025, or 0.0125 mM. To determine the *K_M_* and *V_max_* values for L-tyrosine, a 10 µL aliquot of diluted TH solution was incubated for 1 min at 37 °C with 0.3 mM DMPH_4_ and L-tyrosine at concentrations of 0.8, 0.4, 0.2, 0.1, 0.05, 0.025, or 0.0125 mM. TH activity was assayed as described above (Section 4.8) and expressed as pmol of L-DOPA formed per minute per µg of protein. Four measurements of TH activity were taken for each concentration of L-tyrosine and DMPH_4_ and for both TH forms.

### 4.10. Determination of the IC_50_ Values for Dopamine and 1,2-Dihydroxybenzene

For the *IC*_50_ assay, a 15 µL aliquot of diluted TH solution was incubated for 15 min at 37 °C with 0.3 mM L-tyrosine, 0.3 mM DMPH_4_, and dopamine at concentrations of 50, 25, 12.5, 6.25, 3.125, 1.56, 0.52, 0.26, or 0 µM or 1,2-dihydroxybenzene at concentrations of 80, 40, 20, 10, 5, 2.5, 1.25, 0.624, 0.312, or 0 µM. TH activity was assayed as described above (Section 4.8) and expressed as pmol of L-DOPA formed per minute per µg of protein. For each TH variant, four concentration–response curves were generated for dopamine and eight concentration–response curves were generated for 1,2-dihydroxybenzene.

### 4.11. Assay of Thermal Inactivation (T_50_)

Aliquots of 15 µL of diluted TH solution were preincubated for 2 min at each of seven temperatures (46, 48, 50, 52, 54, 56, and 58 °C) and then placed on ice, while control tubes were not preincubated. TH activity was then assayed as described above (Section 4.8). Six thermal inactivation curves were generated for each TH variant.

### 4.12. Assessment of Thermal Unfolding (T_m_)

The thermal unfolding of the recombinant TH-278R and TH-278H proteins was assessed by differential scanning fluorimetry (DSF) using the ProteOrange fluorescent dye (Lumiprobe, Moscow, Russia). The dye-containing buffer was prepared by adding 20 µL of 0.1 M DTT and 2 µL of ProteOrange dye (5000× concentrate) to 1980 µL of Tris-HCl buffer (50 mM, pH 7.6). The dye-containing buffer was added to the recombinant protein solution (60 µg of either TH-278R or TH-278H) to a final volume of 500 µL. A 50 µL aliquot of the final solution was transferred to a separate PCR tube (0.2 mL). Changes in the fluorescent signal were recorded from 33–80 °C in 1 °C steps (47 cycles) on a CFX96 thermocycler (Bio-Rad, Hercules, CA, USA) using the SYBR Green fluorescence channel. Eight thermal-unfolding curves were generated for each TH variant using the corresponding protein preparation.

### 4.13. Statistics

The *V_max_* and *K_M_* values for the Michaelis–Menten curves were determined using nonlinear regression (Equation (1)), and the resulting values were compared using a Student’s *t*-test.

(1) *V_t_* = (*V_max_* × [*S*])/(*K_M_* + [*S*])

To calculate the *IC*_50_, the fraction of TH activity lost was determined as (*V*_0_ − *V_I_*)/*V*_0_, where *V_I_* is the TH activity at the *I* concentration of inhibitor and *V*_0_ is the TH activity without inhibitor. This fraction was then plotted against *ln*(*I*) according to the following linear equation (Equation (2)):

(2) *ln*(*I*) = *b* × (*V_I_* − *V*_0_)/*V*_0_ + *a*

where *I* is the inhibitor concentration and *b* and *a* are the slope and intercept of the linear regression, respectively. The *IC*_50_ value was calculated by substituting 0.5 for the fraction of activity lost: *IC*_50_ = exp(*b* × 0.5 + *a*).

To calculate the *T*_50_, the fraction of TH activity lost was determined as (*Vc* − *Vt*)/*Vc*, where *Vt* is the residual TH activity after preincubation at a given temperature and *Vc* is the activity of the unheated control. This fraction was then plotted against temperature according to the following linear equation (Equation (3)):

(3) *T* = *b* × [(*Vc* − *Vt*)/*Vc*] + *a*

where *T* is the preincubation temperature and *b* and *a* are the slope and intercept of the linear regression, respectively. The *T*_50_ value was calculated by substituting 0.5 for the fraction of activity lost: *T*_50_ = *b* × 0.5 + *a*.

Similarly, to calculate the *T_m_*, the fraction of fluorescence was determined as *F_t_*/*F_max_*, where *F_t_* is the fluorescence measured at a given temperature and *F_max_* is the maximal fluorescence. This fraction was plotted against temperature using a linear equation (Equation (4)):

(4) *T* = *b* × (*F_t_*/*F_max_*) + *a*

where *T* is the temperature and *b* and *a* are the slope and intercept of the linear regression, respectively. The *T_m_* value was calculated by substituting 0.5 for *F_t_*/*F_max_*: *T_m_* = *b* × 0.5 + *a*.

Note: Thermal unfolding curves are generally described using hyperbolic or logistic functions [17]. However, these curves contain a quasi-linear region corresponding approximately to 0.15–0.86 of the transition, which includes the 0.5 point used to determine *T_m_*. In the present study, the relationship within this quasi-linear region was accurately approximated by linear regression (R^2^ > 0.95). Therefore, the linear approximation was used to calculate *T_m_* as a simpler approach that adequately described the experimental data.

All data are presented as the mean ± SEM and were analyzed using one-way ANOVA and Student’s *t*-test.

## 5. Conclusions

This study characterized the molecular effects of the R278H substitution on the activity and stability of mouse TH. Using recombinant TH-278R and TH-278H forms of mouse TH, we showed that the substitution (1) decreased *V_max_*; (2) did not affect the *K_M_* for the substrate L-tyrosine; (3) increased the *K_M_* for the cofactor DMPH_4_; (4) did not affect the *T*_50_ of thermal inactivation; (5) increased the *T_m_* of thermal unfolding; and (6) decreased the *IC*_50_ values for dopamine and 1,2-dihydroxybenzene.

The lower *IC*_50_ values for dopamine and 1,2-dihydroxybenzene indicate that the R278H substitution increases the inhibitory potency of these catechol-containing compounds toward TH. Given that these compounds share a catechol moiety with the TH product L-DOPA, one possible explanation for the reduced *V_max_* of TH-278H is that the R278H substitution alters the interaction of the enzyme with its product. However, the present experiments provide no direct evidence for this mechanism: exogenous L-DOPA did not inhibit either TH-278R or TH-278H. This mechanism therefore remains viable but unconfirmed. An alternative possibility, consistent with the increased *T_m_* of TH-278H, is that increased structural stability limits the conformational flexibility of the catalytic domain required for optimal catalysis. Further structural and kinetic studies will be required to distinguish between these and other possibilities.

## Data Availability

The original contributions presented in this study are included in the article/Supplementary Materials. Further inquiries can be directed to the corresponding author.

## Supplementary Materials

The following supporting information can be downloaded at https://www.mdpi.com/article/10.3390/ijms27209370/s1.
