# Supplementary material for: R278H Substitution Reduces Mouse Tyrosine Hydroxylase Activity and Increases Susceptibility to Catechol Inhibition
Source: Int J Mol Sci. 2026 Oct 21;27(20):9370. doi: 10.3390/ijms27209370 (PMC13705442; doi:10.3390/ijms27209370)
Supplement: Supplementary file 1 [file ijms-27-09370-s001.zip › ijms-4546340-supplementary.pdf]

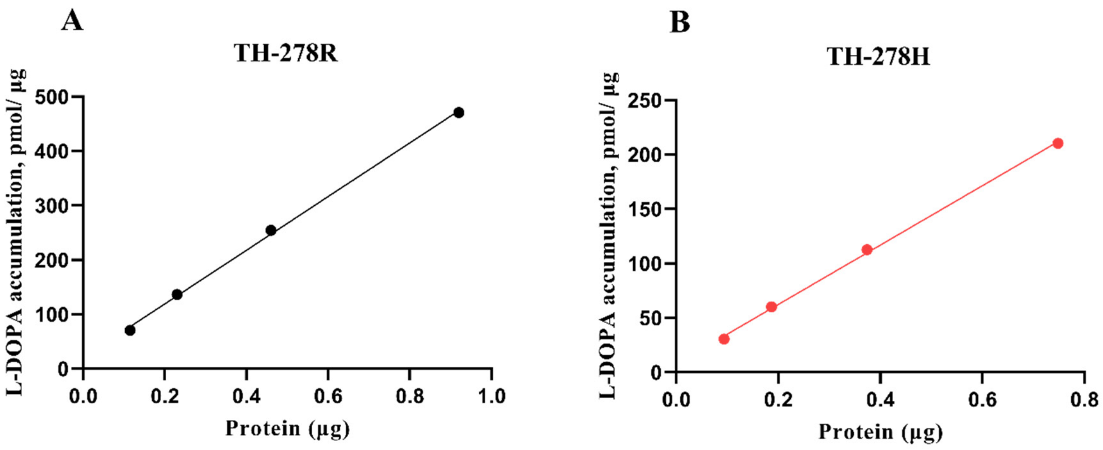

Figure S1. Dependence of L-DOPA accumulation (pmol) after 15 min of incubation on the amount of TH-278R (A) and TH-278H (B) protein (μg). A strong linear relationship was observed for both forms (TH-278R: $R^2 = 0.991$, $n = 24$; TH-278H: $R^2 = 0.988$, $n = 24$).

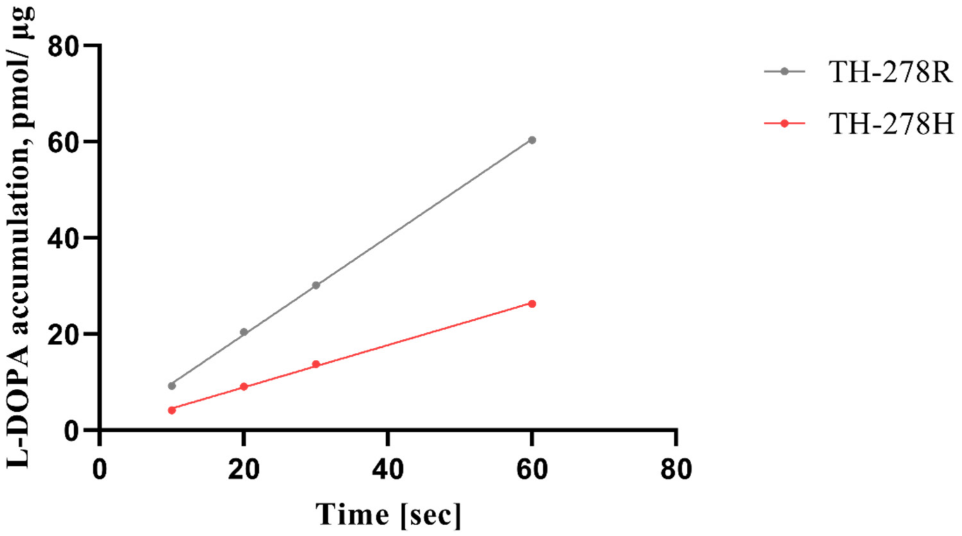

Figure S2. Dependence of L-DOPA accumulation (pmol/μg) on incubation time (s) during the first minute of incubation for the TH-278R and TH-278H forms. The amount of L-DOPA synthesized in the first minute of incubation was higher for TH-278R (60.99 ± 1.11 pmol/μg) than for TH-278H (26.36 ± 0.71 pmol/μg, $t(22) = 26.3$, $p < 0.001$).

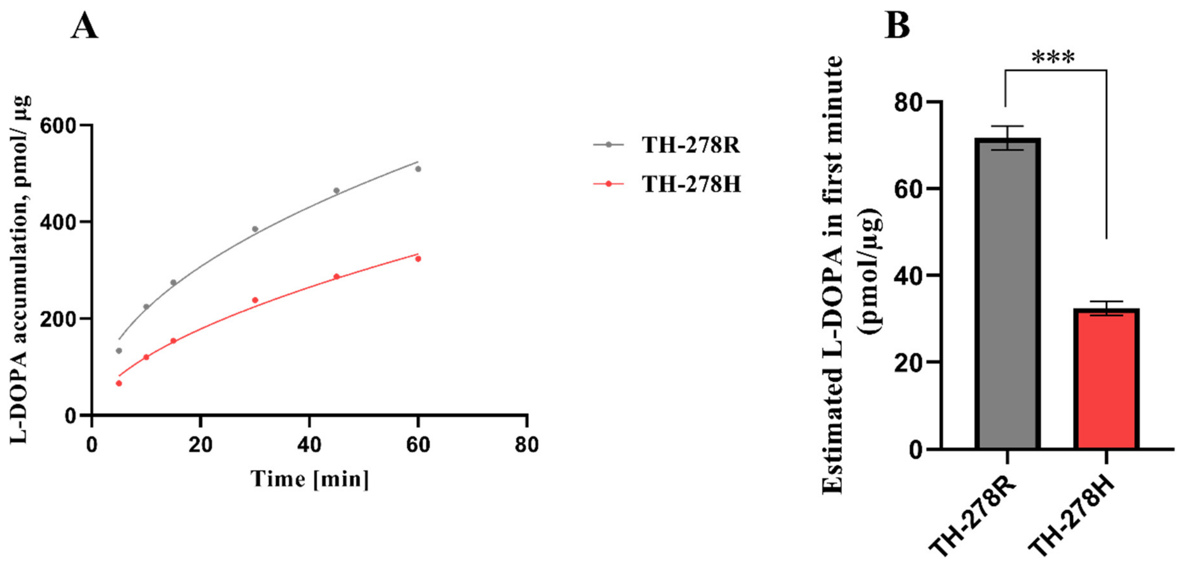

Figure S3. Dependence of L-DOPA accumulation (pmol/µg) on incubation time (min) between 5 and 60 min of incubation for the TH-278R ($R^2 = 0.93$, $n = 48$) and TH-278H ($R^2 = 0.99$, $n = 48$) forms. (A) L-DOPA accumulation (pmol/µg) versus incubation time (min). (B) Amount of L-DOPA synthesized (pmol/µg) in the first minute of incubation, estimated from the curves in (A). ***$p < 0.001$.

Note: To estimate the amount of L-DOPA synthesized in the first minute of incubation, the curves were approximated by a power function, $A_t = A_1 * t^{\alpha}$, where A_t is the amount of L-DOPA synthesized after t min of incubation, A_1 is the amount of L-DOPA synthesized in the first minute of incubation, and α is the power coefficient. The values of α for TH-278R (0.486 ± 0.011, $n = 48$) were lower than those for TH-278H (0.569 ± 0.014, $n = 48$, $t(94) = 4.66$, $p < 0.001$). The values of A_1 for TH-278R (71.68 ± 2.76 pmol/µg, $n = 48$) were higher than those for TH-278H (32.48 ± 1.62 pmol/µg, $n = 48$, $t(94) = 12.28$, $p < 0.001$). These estimated A_1 values are close to the experimentally measured amounts of L-DOPA synthesized in the first minute of incubation (see Figure S2).

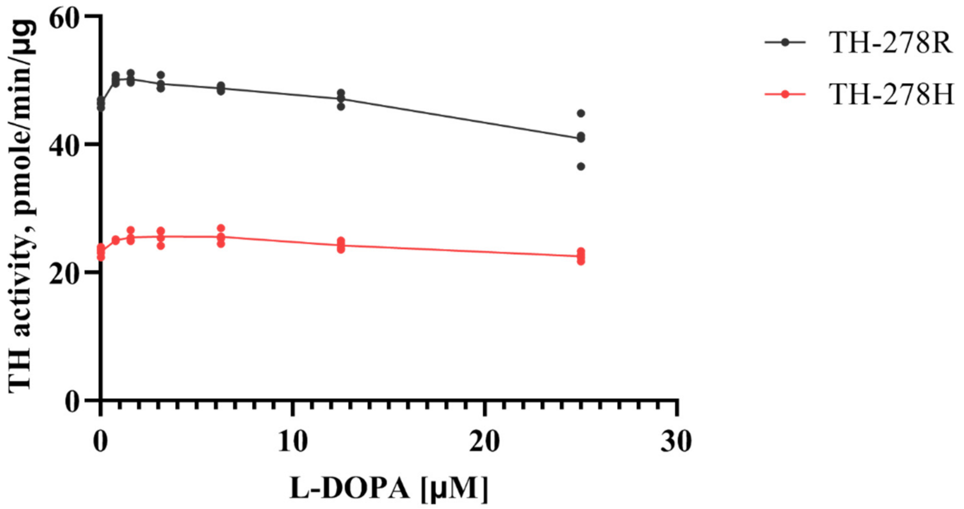

Figure S4. Activity of the TH-278R and TH-278H forms in the presence of different concentrations of exogenous L-DOPA.

Note: Exogenous L-DOPA did not inhibit either TH-278R or TH-278H.
